# Supplementary material for: The Impact of Norepinephrine on Mono-Species and Dual-Species Staphylococcal Biofilms
Source: Microorganisms. 2021 Apr 13;9(4):820. doi: 10.3390/microorganisms9040820 (PMC8070549; doi:10.3390/microorganisms9040820)
Supplement: Supplementary file 1 [file microorganisms-09-00820-s001.zip › Table S1.docx]

| Kinetic parameters | Planktonic growth in model I, aerobic | | Biofilm growth in model II,  aerobic | | Planktonic growth in model I, anaerobic | | Biofilm growth in model II,  anaerobic | |
| --- | --- | --- | --- | --- | --- | --- | --- | --- |
|  | Control | NE 3.5×10^-7^ | Control | NE 3.5×10^-7^ | Control | NE 3.5×10^-7^ | Control | NE 3.5×10^-7^ |
| *S aureus* 209P | | | | | | | | |
| Maximal OD _540_ | 1.39 | 1.38 | 1.01 | 0.98 | 1.25 | 1.3 | 1.01 | 1.15 |
| Specific growth rate, h^-1^ | 0.27 | 0.32 | 0.21 | 0.19 | 0.26 | 0.28 | 0.22 | 0.22 |
| Doubling time, h | 2.61 | 2.48 | 3.28 | 3.5 | 2.67 | 2.48 | 3.20 | 3.21 |
| Linear portion of the curve, h | 4.25 | 3.5 | 7.00 | 6.50 | 4.50 | 4.25 | 4.00 | 4.75 |
| *S epidermidis* ATCC 14990 | | | | | | | | |
| Maximal growth (OD _540_) | 1.58 | 1.61 | 1.01 | 1.11 | 1.43 | 1.43 | 1.17 | 1.07 |
| Specific growth rate, h^-1^ | 0.28 | 0.30 | 0.29 | 0.28 | 0.32 | 0.33 | 0.27 | 0.25 |
| Doubling time, h | 2.46 | 2.30 | 2.42 | 2.52 | 2.20 | 2.1 | 2.55 | 2.81 |
| Linear portion of the curve, h | 4.5 | 3.25 | 3.50 | 5.25 | 4.25 | 4.00 | 4.00 | 4.50 |
| Binary culture | | | | | | | | |
| Maximal growth (OD _540_) | 1.52 | 1.51 | 1.08 | 1.05 | 1.34 | 1.29 | 1.11 | 1.15 |
| Specific growth rate, h^-1^ | 0.34 | 0.33 | 0.26 | 0.24 | 0.3 | 0.29 | 0.27 | 0.26 |
| Doubling time, h | 2.04 | 2.07 | 2.65 | 2.83 | 2.3 | 2.37 | 2.60 | 2.71 |
| Linear portion of the curve, h | 3.50 | 2.50 | 6.00 | 5.50 | 4.25 | 4.25 | 3.75 | 4.00 |

Table S1: Kinetic parameters of growth curves in two different models
