# Supplementary figures and images for: The Impact of Norepinephrine on Mono-Species and Dual-Species Staphylococcal Biofilms
Source: Microorganisms. 2021 Apr 13;9(4):820. doi: 10.3390/microorganisms9040820 (PMC8070549; doi:10.3390/microorganisms9040820)

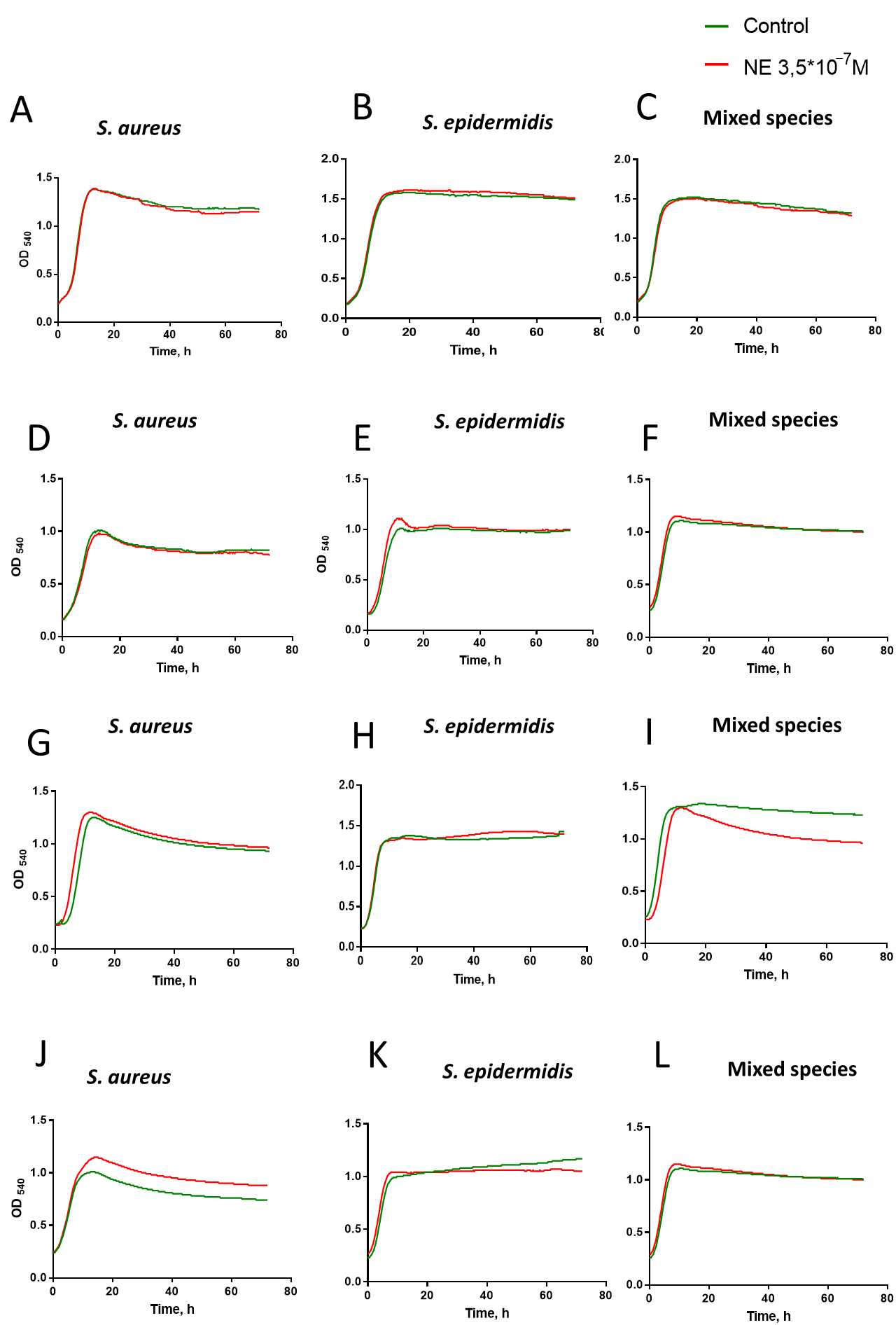

Supplement: Supplementary file 1 [file microorganisms-09-00820-s001.zip › Fig S1.tif]
